# Lurasidone treatment in a delusional disorder patient with atrial fibrillation: A case report

**DOI:** 10.1192/j.eurpsy.2021.2103

**Published:** 2021-08-13

**Authors:** S. Marini, I. Matarazzo, A. Gentile

**Affiliations:** Centro Di Salute Mentale, AsreM, Termoli, Italy

**Keywords:** Delusional Delirium, lurasidone, Atrial Fibrillation

## Abstract

**Introduction:**

Psychosis itself may be associated with an increased risk of atrial fibrillation. Moreover, antipscyhotic treatment increases this risk. Recently D’Urso et al. reported aripiprazole-induced atrial fibrillation in a patient with concomitant risk factors.

**Objectives:**

To the best of author’s knowledge no data has been published about the safety and the efficacy of lurasidone treatment in psychotic patient with comorbid atrial fibrillation.

**Methods:**

A 68 years old patient with persistent atrial fibrillation and hypertension in treatment with amiodarone 100 mg/day, lurasidone 25 mg/day, rivaroxaban 15 mg/day, clopidogrel 75 mg/day, bisoprolol 1,25 mg/day, tamsulosin 0,4 mg/day presented delusional ideas of jealousy for not real betrayal by his wife, social withdrawal, reduced sleep. Blood pressure 130/80 mmHg, heart rate 70 bpm, Qtc 420 msec. The patient was drug-naïve for any psychotropic treatment. The authors decided to start lurasidone treatment at the dosage of 18,5 mg/day.

**Results:**

After the first administration of lurasidone treatment sleep was resolved. After two weeks delusional ideas and social withdrawal were markedly improved. After one month of treatment, blood pressure, heart rate and Qtc remained almost stable. The authors decided not to increase the dosage of lurasidone because of the age of the patient and the comorbid cardiovascular pathologies.

**Conclusions:**

Lurasidone has showed safety and efficacy in the treatment of this patient with delusional disorder and comorbid atrial fibrillation. This is a preliminary data that requires follow up and further studies to confirm the usefulness of lurasidone in psychotic patients with atrial fibrillation and cardiovascular risks.

**Disclosure:**

No significant relationships.

